# Extravasation of contrast medium during CT examination: an observational case-control study

**DOI:** 10.11604/pamj.2015.20.89.3276

**Published:** 2015-01-30

**Authors:** Zayneb Alami, Siham Nasri, Samir Ahid, Hanane Hadj Kacem

**Affiliations:** 1 Pharmacovigilance Unit, Mohamed VI University Hospital, Department of Pharmacology, Medical and Pharmacy School, University Mohammed the First, Oujda, Morocco; 2 Department of Radiology, Mohamed VI University Hospital, Medical and Pharmacy School, University Mohammed the First, Oujda, Morocco; 3 Pharmacoepidemiology and Pharmacoeconomics Research Team. Mohammed V Rabat University, Morocco

**Keywords:** Extravasation, contrast media/medium, computed tomography (CT), risk factors

## Abstract

**Introduction:**

Extravasation is an adverse reaction to intravenous injection of contrast medium (CM) during CT examination. The objectives of this study are to determine the frequency, management and outcomes of extravasations and to assess risk factors for extravasation.

**Methods:**

Every incident of extravasation which occurred between March 2012 and March 31, 2013 was recorded in an extravasation form. Ethics Committee approval was obtained and the patients gave their consent to participate in the study. Data collected in the form included patients’ age, sex, comorbidities, symptoms, CM used, injection mode, site and rate, extravasated volume, location of extravasation, severity of injury, treatment and patient outcome. Each case was matched with 4 controls of the same age ± 5 years and the same gender when possible.

**Results:**

Extravasation occurred in 18 (7 women, 11 men) out of 2,000 injections of CM (0.9%) with a median age of 53 (10-78) years. Automated injection was performed in all cases with a mean rate of 1.7ml/s. Large extravasated volumes (≥ 50ml) were more observed in patients undergoing CT angiography (28.6% vs. 6.6%, although not significant *P=0.112*). Multivariate analysis revealed a significant association between patients with cardiac diseases and extravasation (OR: 7.3, 95% CI (1.09-49.05), P=0.04) whereas the injection rate is a protective factor from extravasation (*P=0.002*).

**Conclusion:**

Extravasation of CM results in mild to moderate adverse effects in all cases. Our study suggests that patients with cardiac disease are more predisposed to contrast extravasation than others. Further and larger studies are needed to confirm this trend.

## Introduction

Contrast media (CM) are widely used in in computed tomography (CT) examination. CM may lead to various adverse reactions that may occur immediately (in the hour following the injection) or later (up to a week after the injection). The possible adverse reactions are an extravasation of CM, allergic, cardiovascular or renal reactions [1]. Extravasation, i.e. the flow of CM out of the vein into which it was injected, is a well-recognized adverse reaction to intravenous injection of CM in CT units. The main mechanism involved in extravasation is excessive pressure in the injection line mainly occurring with a high osmolality level, above 1.025-1.420 mOsm/kg water. Extravasation may be the result of the cytotoxicity of CM, its ionic character or not with conflicting results reported in the literature, the presence of meglumine as the cation, the volume of extravasated CM and the mechanical compression caused by large-volumes and indwelling intravenous lines [2]. When extravasation occurs from indwelling intravenous lines, it is often due to the development of phlebitis in the vein that is canulated [3]. A higher risk of extravasation is reported not only with elderly patients and small children, but also with unconscious and debilitated patients who are unable to complain about pain. Extravasation injury seems to be more severe in patients with subcutaneous atrophy, arterial insufficiency (due to atherosclerosis, diabetes mellitus or connective tissue diseases), or impaired venous or lymphatic drainage. Cancer patients receiving radiotherapy and/or chemotherapy are also considered at high risk because of poor lymphatic drainage, fragile and small-caliber veins [2–5]. Fortunately, extravasation mostly results in minor signs: redness, swelling, localized erythema and pain. In few cases, extravasation is severe with skin blistering, tissue necrosis and rarely, a compartment syndrome [4, 5]. According to the literature, incidence of extravasation is ranging from 0.03% to 0.94% [6–15]. The aim of this study was to determine the frequency, management and outcome of extravasation of CM in our hospital and to assess the possible risk factors of extravasation by a case-control study.

## Methods

### Population and study design

During a one-year period, between March 2012 and March 31, 2013, data for each extravasation incident observed after CM injection were prospectively recorded in an extravasation form. The forms were completed by a radiology resident. Data obtained included patient sex, age, comorbidity, type of CM extravasated, injection site, intravenous catheter gauge, contrast material injection rate, estimated extravasated volume (EEV), patient symptoms, severity of injury and treatment. Severity of injury was determined by initial signs reported and evolution. The study has Ethics Committee approval and the patients gave their consent to participate in the study.

### Inclusion criteria

Each patient who developed an extravasation incident during CT examination with CM injection during the study period was considered as a case. Each case was matched with 4 controls so as to achieve a statistical power of 80%. The controls were patients with no extravasation but were blindly selectionned with the following inclusion criteria's: same age ± 5 years and same gender when possible.

### Contrast medium (CM)

CM used during this period in the CT unit: ioxitalamate (Telebrix^®^ 35, Guerbet, France), Iopromide (Ultravist^®^, Bayer Schering Pharma AG Germany) and iobitridol (Xenetix^®^, Guerbet, France).

### Injection technique

Injections were performed by nurses experienced in intravenous injection technique. In most cases, an automated injector (Nemoto) was used and whenever it failed, we used manual injection. The injection site usually used was the antecubital fossa. If that venous access was not established, we used the vein in the forearm or in the hand. Lower extremities are never used in our hospital. In adults, we usually use 22-(blue) or 20-(pink) gauge catheters, rarely 18-(green) gauge catheters whereas in children, the 24-(yellow) gauge catheter is preferred. In hospitalized patients with indwelling intravenous catheters, patency is checked with a saline injection before starting the CM injection. Injection site is monitored in all patients from before initiation of CM injection until scan acquisition is complete. Adverse reactions to CM are well explained to patients, including instructions to report any pain or discomfort. Lacking plastic surgery in our hospital, a traumatology or vascular surgery consultation was provided for all extravasation injuries. Only clinical signs and symptoms were assessed to establish the severity of extravasation.

### Statistical analysis

The statistical analysis was performed using the Statistical Package for Social Sciences (SPSS) v13.0. (Inc. Chicago,IL). The results were expressed as mean±standard deviation or in count and percentage. The comparison of quantitative and qualitative variables was carried out using respectively Student's t-test, Chi-square test and the exact Fisher test when the Chi-square was not applicable. A threshold of P<0.05 was considered significant. The variables collected from the study were tested for their potential relationship with extravasation. In a first step, each variable was assessed independently by univariate analysis. In a second one, the variables with P<0.05 were fed into a multiple regression model to assess the odds ratio.

## Results

**Figure 1:**
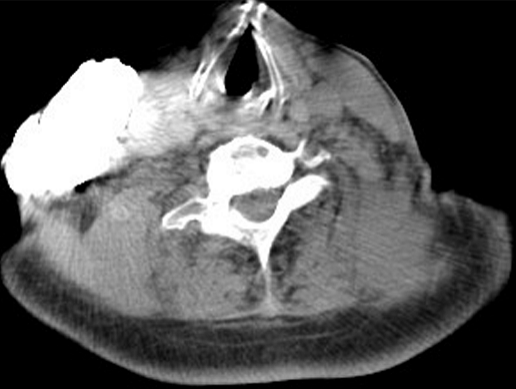
Axial contrast-enhanced chest CT scan demonstrates extravasation of non- ionic CM into the right subcutaneous cervical region

**Figure 2:**
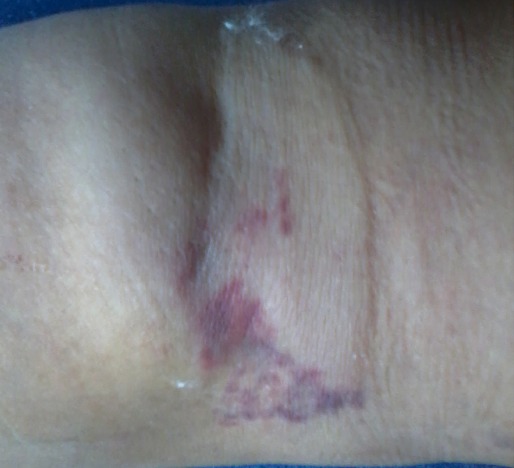
Moderate extravasation injury in an adult patient demonstrates swelling and ecchymosis in the antecubital fossa

Between March 2012 and March 31, 2013, there were 7,461 CT examinations. Two thousands of them required intravenous injections of CM in our hospital (some patients underwent more than one CT examination). Extravasation occurred in 18 out of 2,000 injections (0.9%): 7 women and 11 men (Sex ratio M/F: 1,57) with a median age of 53 years (10-78 years). In three cases, extravasation occurred in a child (10yrs.) and 2 young adults (20, 26 yrs). Cardiovascular diseases (n=7) and neoplastic disorders (n=4) were the most common comorbidities in our patients. An automated injector was used in all patients with an injection rate between 1.5 and 2ml/s and a mean rate of 1.7 ml/s. Catheters used for contrast material injection were: 20 gauge (n=11), 22 gauge (n=4) and 18 gauge (n=2). The injection sites were located in the antecubital fossa in 10 cases, the dorsum of the hand (n=4) and wrist (n=3) and jugular vein in one case. Ionic and high-osmolality agents were used in only 4 cases. Five patients had an infusion for more than 24h. In 6 cases, the injections were upstream from a recent puncture site. In four patients of the 18 cases, acquisition was performed using a CT angiography protocol. Non-ionic extravasation volumes ranged from 10 to 100 ml in 15 patients (Figure 1). In three cases, the extravasated volume was not mentioned. Common symptoms reported were pain, swelling and edema (Figure 2, Table 1). Treatment consisted of suction of extravasated CM volume, application of ice packs, alcoholized bandage and elevation of the upper extremity. An additional treatment using painkillers based on the association of paracetamol and codeine was administered in 8 cases and local corticosteroid injection in the extravasation site in 4 cases. A traumatology or vascular surgery consultation was performed for 15 patients. None of the 18 cases was life threatening; all patients were asymptomatic after 48h, except for one patient who died of his comorbidity. We compared data from patients who experienced extravasation with data from a control sample of 72 patients who underwent IV injection of CM but had no complications of IV CM injection (Table 2).There is no difference between the two groups of age, gender or comorbidities except of cardiac diseases (33.3% vs. 8.3% in the control group, *P=0.02*). Automated injector was used in all patients. Ionic and high osmolality agents were more used in patients with extravasation than in the control group (*P=0.001*). No difference statistically significant in the injection site was observed between the two groups whereas the injection rates of CM were different (1.78 vs 1.93 ml/sec in the control group, Table 3 show that cardiac diseases increase the risks for 7.3 fold (95% CI (1.09-49.05) which is a statistically significant increase (*P = 0.04*) whereas the injection rate is a protective factor from extravasation (*P=0.002*).

**Table 1:** Description of extravasation injuries observed in patients

No.	Age (y)	Sex	Comorbidities	Type of CM	CT Angiography	EEV (ml)	Location of Extravasation	Symptoms
1	75	M	Hypertension	NI	Yes	100	AF	Blister, swelling, pain
2	78	F	Myocardial ischemia	NI	Yes	90	AF	Pain, edema, ecchymosis
3	75	M	Hypertension	NI	Yes	90	J	Cervical swelling
4	47	F	None	NI	No	80	AF	Pain, swelling,ecchymosis
5	39	F	Dyslipidemia, Breast fibroadenoma	NI	No	76	AF	Swelling
6	48	M	Asthma	NI	No	50	AF	Pain, edema, redness
7	55	F	Neoplastic disorder	NI	No	40	W	Swelling
8	20	F	Myocardial ischemia	I	No	30	AF	Pain, swelling, edema in biceps, hyperchromic skin lesions
9	72	M	None	I	No	30	AF	Pain, edema, redness
10	10	M	Pyschomotor retardation	NI	No	21	AF	Pain, swelling
11	46	F	Breast cancer	NI	No	20	W	Pain, edema
12	52	M	None	NI	No	10	W	Pain,edema, redness
13	54	M	None	NI	No	10	AF	Pain, edema
14	26	M	Brain cancer	NI	No	10	D	Pain, swelling
15	66	M	Stomach cancer	I	No	*	D	Pain, edema, inflammation
16	61	M	Diabetes mellitus, hypertension	NI	No	<30	D	Pain, edema
17	42	F	Valve replacement, Lymph node tuberculosis	I	No	*	D	Pain, redness, edema
18	66	M	Rhythm disorder, vasculitis	NI	Yes	*	AF	Pain, redness

* Not available

EEV: Estimated extravasated volume

I: ionic, NI: non-ionic, AF: antecubital fossa, J: jugular vein, W: wrist, D: dorsum of hand

**Table 2:** Characteristics of the population

Characteristics	Extravasation n=18	Controls n=72	*P-value*
**Age (years)**	51.8 ± 19.4	51.3 ± 19.3	*0.92*
**Sex:** *Male*	11 (61.1%)	37 (51.4%)	*0.46*
**Comorbidities**			
*Cardiac disease*	6 (33.3%)	7 (9.7%)	***0.02***
*Diabetes mellitus*	1 (5.6%)	3 (4.2%)	*0.60*
*Asthma*	1 (5.6%)	2 (2.8%)	*0.49*
*Neoplastic disease*	4 (22.2%)	30 (41.7%)	*0.13*
**Contrast medium** *Ionic* *High Osmolality*			
	4 (22%)	-	***0.001***
	4 (25%)	12 (75%)	***0.001***
**CT Angiography**	3 (17.6%)	4 (7.8%)	*0.35*
**Injection rate (ml/s)**	1.78 ± 0.17	1.93 ± 0.14	***< 0.001***
**Injection site**			
*Antecubital fossa* Other^*^	8 (44.4%) 10 (55.6%)	32 (45.7%) 38 (54.3%)	*0.92*
**Intravenous catheter Gauge**			
*16 Gauge*	1 (5.6%)	1 (1.4%)	*0.365*
*18 Gauge*	2 (11.1%)	24 (33.8%)	*0.059*
*20 Gauge*	11 (61.1%)	1 (1.4%)	***< 0.001***
*22 Gauge*	4 (22.2%)	45 (63.4%)	***0.003***
**Injection upstream from a recent puncture site**	6 (40%)	9 (34.6%)	*0.73*
**Infusion > 24h**	5 (38.5%)	7 (36.8%)	*1*

* Dorsum of hand, wrist, jugular vein

**Table 3:** Risk factors for extravasation analyzed by univariate and multivariate analysis

	Univariate analysis			Multivariate analysis		
Variables	Odds Ratio	CI 95%	P-value	Odds Ratio	CI 95%	P-value
Comorbidities	1.49	[0.52–4.27]	*0.46*			
*Cardiac disease*	4.64	[1.32–16.24]	***0.016***	7.31	[1.09-49.05]	***0.04***
*Diabetes mellitus*	1.35	[0.13–13.83]	*0.80*			
*Asthma*	2.06	[0.18-24.05]	*0.56*			
*Neoplastic disease*	0.40	[0.12–1.34]	*0.14*			
*CT Angiography*	2.52	[0.50–12.61]	*0.26*			
*Injection rate*	0.002	[0–0.70]	***0.001***	0	[0-0.05]	***0.002***
*Injection site*	1.05	[0.37–2.98]	*0.92*			

## Discussion

This is the first study to estimate the rate of extravasation in our country. The overall extravasation rate in our institution (0.9%) is similar to the rates reported in the literature [6–15] (Table 4). However, the short period of our study (one year) and our sample size (2,000 in comparison to the largest study of *Wang et al*. [13] about intravenous injections) are important limitations of our study. In our study, extravasation has occurred when injections were applied on the dorsum of hand and the wrist (55.6% vs.54.3% in the control group) but also when injection was performed in the antecubital fossa with no difference between the two groups (44.4% vs.45.7% in the control group; *P=0.92*). This result is in contrast with other results, comparing 51 patients with extravasation with 100 patients who did not, reporting a higher percentage of extravasation in patients that have been injected outside the antecubital fossa (39% vs.24%) [16].This may be due to the fact that intravenous access in the antecubital fossa was difficult in our patients, as it was the case in the study of *Wang et al*. [13]. Sixty one percent (61.1%) of patients who had extravasation were injected through a 20-gauge catheter with a statistically significant difference with the control group (1.4%, *P <0.001*), even in respect of the catheter gauge recommendations. In the control group, most patients were injected through a 22-gauge catheter (63.4% vs. 22.2%; *P=0.003*). This finding doesn't seem to be in line with the results of Wienbeck et al. [15] where 4,457 patients were prospectively studied. We acknowledge that the sample size is a great limitation of our study.

**Table 4:** Rate of CM extravasation in published series

Authors [Ref]	Year Published	Sample size	Type of population	Type of CM	No of extravasations	Rate (%)
Cohan et al [6]	1990	14,000	Adult and children	Non-ionic	5	0.03
Miles et al [7]	1990	5,280	Adult and children over 12y.	Ionic, Non-ionic	6	0.10
Sistrom et al [8]	1991	20,950	Adult and children	Non-ionic	28	0.13
Cohan et al [9]	1997	22,254	Adult and children	Ionic and Non-ionic	51	0.23
Federle et al [10]	1998	5,106	Adult	Ionic, Non-ionic	48	0.94
Jacobs et al [11]	1998	6,660	Adult and children	Ionic, Non- ionic	40	0.60
Cochran et al [12]	2002	66,029	Adult and children	Non-ionic	225	0.34
Wang et al [13]	2007	69,657	Adult and children	Non-ionic	475	0.70
Callahan et al [14]	2009	12,494	Children	Non-ionic	57	0.46
Wienbeck et al [15]	2010	4,457	Adult	Non-ionic	52	1.2
**Total**		**226,887**			**950**	**0.42**

The extravasated volume of CM observed in our study ranged from 10 to 100ml. The largest study of *Wang et al*. [13] has reported an EEV varying from less than 10ml to 150ml. Large volumes of extravasation (90 and 100ml) were noted in patients who received a dynamic bolus CT injection located in the antecubital fossa and in the jugular in one case. In fact, dynamic bolus CT induces large volumes of CM [4]. A large volume extravasation may be also observed when extravasation is deep and/or when the patient remains asymptomatic [2]. The smallest volume (10ml) was observed when the injection was performed in the wrist or dorsum of the hand. Probably, the small surface areas of these portions of the limb limit the diffusion and the extravasation of CM. Large volumes (EEV ≥ 50ml) were observed in patients undergoing CT angiography (28.6% vs. 6.6%). However, this difference was not statistically significant (*P=0.112*). This can be explained by only 7 cases of CT angiography, probably not sufficient enough to prove this relationship. Because automated injector was used in all patients, we couldn't analyze its influence on extravasation. However, it is recognized that automated power injectors increase the flow of mechanical CM administration. The risk is higher with a “bolus-tracking” technique used in CT angiography [6]. In this study, CT angiography was more used in patients who had extravasation than in the control group (17.6% vs. 7.8%) but the difference was not statistically significant (*P=0.35*). This can be also explained by the few cases of CT angiography. As injection rate was always between 1.5 and 2ml/s in all patients, we could not analyze the influence of injection flow rate and extravasation. In the current study, there was no severe extravasation. Only mild to moderate extravasations of CM were recorded. This might be explained by the low flow rate applied and by our sample size that is too small to evaluate the rates of different severities of injuries. Extravasation was treated by suction of extravasated CM volume, application of ice packs, alcoholized bandage, elevation of the upper extremity and administration of painkillers and local corticosteroid injections. According to current guidelines [2, 4], there is no experimental evidence or consensus for the treatments used but conservative management is often sufficient without reconstructive surgery [6]. Administration of painkillers or local corticosteroid injection in most cases of our patients was not justified. Thus, management of extravasation in our institution must be reviewed and changed. We had written a form explaining the strategy of extravasation management. The form was presented to all staff of the Department of Radiology.

## Conclusion

A low Injection rate is a protective factor from extravasation. However, it is not the only one. Our study suggests that patients with cardiac disease are more predisposed to contrast extravasation than others. This is in accordance with the fact that cardiovascular diseases enhance vascular fragility. To our knowledge, none of the published studies analyzed the influence of comorbidities of patients. Further and larger studies are needed to confirm this trend.
